# Dual color fluorescence *in situ* hybridization (FISH) assays for detecting *Mycobacterium tuberculosis* and *Mycobacterium avium* complexes and related pathogens in cultures

**DOI:** 10.1371/journal.pone.0174989

**Published:** 2017-04-11

**Authors:** Jyotsna Shah, Helena Weltman, Patricia Narciso, Christina Murphy, Akhila Poruri, Shrikala Baliga, Leesha Sharon, Mary York, Gail Cunningham, Steve Miller, Luz Caviedes, Robert Gilman, Edward Desmond, Ranjan Ramasamy

**Affiliations:** 1 ID-FISH Technology Inc., Palo Alto, California, United States of America; 2 Kasturba Medical College, Mangalore, Manipal University, India; 3 University of California, San Francisco, California, United States of America; 4 Labotorio de Investigación en Enfermedades Infecciosas of the Universidad Peruana Cayetano Heredia, Iquitos, Peru; 5 John Hopkins University, Baltimore, Maryland, United States of America; 6 Microbial Disease Laboratory, California Department of Public Health, Richmond, California, United States of America; Institut de Pharmacologie et de Biologie Structurale, FRANCE

## Abstract

Two rapid dual color fluorescence *in situ* hybridization (FISH) assays were evaluated for detecting *M*. *tuberculosis* and related pathogens in cultures. The MN Genus-MTBC FISH assay uses an orange fluorescent probe specific for the *Mycobacterium tuberculosis* complex (MTBC) and a green fluorescent probe specific for the *Mycobacterium* and *Nocardia* genera (MN Genus) to detect and distinguish MTBC from other Mycobacteria and Nocardia. A complementary MTBC-MAC FISH assay uses green and orange fluorescent probes specific for the MTBC and *M*. *avium* complex (MAC) respectively to identify and differentiate the two species complexes. The assays are performed on acid-fast staining bacteria from liquid or solid cultures in less than two hours. Forty-three of 44 reference mycobacterial isolates were correctly identified by the MN Genus-specific probe as *Mycobacterium* species, with six of these correctly identified as MTBC with the MTBC-specific probe and 14 correctly as MAC by the MAC-specific probe. Of the 25 reference isolates of clinically relevant pathogens of other genera tested, only four isolates representing two species of *Corynebacterium* gave a positive signal with the MN Genus probe. None of these 25 isolates were detected by the MTBC and MAC specific probes. A total of 248 cultures of clinical mycobacterial isolates originating in India, Peru and the USA were also tested by FISH assays. DNA sequence of a part of the 23S ribosomal RNA gene amplified by PCR was obtained from 243 of the 248 clinical isolates. All 243 were confirmed by DNA sequencing as *Mycobacterium* species, with 157 and 50 of these identified as belonging to the MTBC and the MAC, respectively. The accuracy of the MN Genus-, MTBC-and MAC -specific probes in identifying these 243 cultures in relation to their DNA sequence-based identification was 100%. All ten isolates of *Nocardia*, (three reference strains and seven clinical isolates) tested were detected by the MN Genus-specific probe but not the MTBC- or MAC-specific probes. The limit of detection for *M*. *tuberculosis* was determined to be 5.1x10^4^ cfu per ml and for *M*. *avium* 1.5x10^4^ cfu per ml in liquid cultures with the respective MTBC- and MAC-specific probes in both the MN Genus-MTBC and MTBC-MAC FISH assays. The only specialized equipment needed for the FISH assays is a standard light microscope fitted with a LED light source and appropriate filters. The two FISH assays meet an important diagnostic need in peripheral laboratories of resource-limited tuberculosis-endemic countries.

## Introduction

The World Health Organization (WHO) estimates that one third of the human population is infected with *Mycobacterium tuberculosis* (MTB), the causative agent of tuberculosis [1]. A small proportion of those infected, estimated to be 10.4 million new cases in 2015, then develop active disease resulting in 1.4 million deaths in 2015 [1]. An additional 0.4 million deaths from tuberculosis among persons with HIV infection was also reported in 2015 [1]. While most human pulmonary mycobacterial infections are caused by MTB and closely related species such as *Mycobacterium bovis* that together comprise the *Mycobacterium tuberculosis* complex or MTBC, clinically relevant infections with non-tuberculous mycobacteria (NTM) also occur in the USA and elsewhere [2, 3]. The management and treatment of patients with tuberculosis varies from that of patients infected with NTM. Pulmonary disease, the most commonly reported localized manifestation for NTM infections, is most often associated with mycobacterial species related to *Mycobacterium avium* (the *M*. *avium* complex or MAC), but other *Mycobacteria* such as *M*. *kansasii*, *M*. *fortuitum*, *M*. *xenopi*, *M*. *abscessus*, and *M*. *simiae* can be responsible in some instances [4–8]. Although NTM-associated pulmonary disease has been described primarily among immunocompromised persons [2, 3], it is now recognized in some persons without predisposing conditions [5–8]. Norcardiosis caused by *Norcadia* species also needs to be differentiated from MTB in human lung disease [9]. *Norcadia* are Gram positive, partially acid fast-staining bacteria closely related to the genus *Mycobacterium*. They are found in soil and water and norcardiosis can manifest in pulmonary, cutaneous or disseminated forms [9]. An estimated 500–1000 new cases of nocardiosis are diagnosed each year in the USA, with approximately 60% of cases being associated with immune-compromise, *e*.*g*. due to HIV infection, cancer and diabetes [9].

The identification of MTB, following a presumption of tuberculosis on clinical criteria, conventionally relies on microscopic examination for acid-fast staining bacilli, *e*.*g*. with the Ziehl-Neelsen stain, in sputum or tissue [10]. However, the acid-fast staining procedure detects all species of mycobacteria and lacks sufficient sensitivity with sputum smears and tissue samples. The Xpert^®^ MTB/RIF system (Cepheid, Sunnyvale, CA, USA), a test based on PCR amplification and detection of the relevant DNA sequences in MTB, is recommended by the WHO as an initial diagnostic test for MTB and rifampicin resistance in the sputum of adults and children presumed to have tuberculosis [11–13]. Although about 100 times more sensitive in detecting MTB than conventional acid-fast stained smear microscopy, Xpert® MTB/RIF presently identifies only MTB, and its cost limits its use in resource-limited countries. Culturing from clinical specimens therefore retains an important role in correctly identifying the causative pathogen in tuberculosis-like disease, particularly in the case of smear negative, pediatric or extra pulmonary infections and in peripheral laboratory settings in resource-limited countries. A variety of liquid and solid culture formats that significantly reduce culture times are now available for mycobacteria [14]. The correct identification of mycobacteria from cultures at the species level frequently relies on observing colony characteristics [14], and additional biochemical, immunological or molecular biological tests [12–14]. The various molecular tests based on nucleic acid amplification or multiple chemiluminescent probes currently available for culture characterization ([12–14] S1 Table) are relatively expensive and therefore of limited value in many tuberculosis-endemic countries that often lack the requisite resources. Immunochromatographic tests to detect specific proteins produced by MTBC are more cost-effective, but have specificity and sensitivity constraints ([12–14] S1 Table). Appropriate new tests that can simultaneously identify and differentiate MTBC, MAC, non-MAC NTM and *Norcardia* species in clinical samples and cultures derived from them in resource-limited countries can therefore enhance the worldwide effort to control tuberculosis [11–14].

Fluorescence *in situ* hybridization (FISH) is a cytogenetic technique that utilizes short complementary probes labeled with fluorescent molecules to localize and detect specific target DNA or RNA sequences. Delong *et al*. demonstrated in 1989 that FISH can be used to detect a single microbial cell using fluorescently labeled oligodeoxynucleotides complementary to 16S ribosomal RNA (rRNA), and that with appropriately-targeted probes labeled with different fluorescent dyes, FISH can distinguish closely related organisms [15]. The presence of multiple copies of rRNA in the cytoplasm enables the reaction with specific probes to be visualized without PCR amplification of the target sequence. Several FISH tests based on DNA probes [16, 17] or peptide nucleic acid probes (PNA, where the sugar phosphate backbone is replaced with a polyamide chain) [18–20] directed against rRNA to detect MTB and some species of NTM in cultures have been described. PNA FISH probes have been used to differentiate MTBC from NTM in acid-fast bacillus-positive cultures [18]. The MTBC-specific PNA probe in this study [18] had a sensitivity of 84–97% and specificity of 100%. The NTM-specific PNA probe had a sensitivity of 64–91% and specificity of 100%. This test was approved for culture confirmation in Europe. An *M*. *avium*-specific PNA FISH probe has also been applied to detect *M*. *avium* in water samples and biofilms [19]. The different FISH assays for tuberculosis developed to date remain to be tested against a wide range of clinical isolates and are not in common use due to issues with their sensitivity [20] and cost. The advantages of PNA probes over DNA probes reportedly include more favorable stability, cell wall penetration and hybridization characteristics [20]. We recently described the use of DNA probe-based FISH assays with good stability, membrane penetration and hybridization properties that are suitable for use in resource-limited malaria-endemic countries to specifically identify the human malaria parasites *Plasmodium falciparum* and *Plasmodium vivax* [21]. Diagnostic tests for tuberculosis need to be similarly compatible with the epidemiological situation, existing laboratory networks and available resources in disease-endemic countries [12–14]. We report here on two sensitive and specific DNA probe-based dual fluorescence FISH assays that when used in combination rapidly detect and differentiate MTBC, MAC and other NTM/*Nocardia* from liquid and solid cultures, and which also meet the criteria for application in resource-limited, tuberculosis-endemic countries.

## Materials and methods

### Reference cultures

Forty-four clinically relevant reference cultures of *Mycobacterium* species and three of *Nocardia* species (details provided in Table 1 under Results) were used for validating the FISH assays. An additional reference collection of 25 different clinically relevant pathogen cultures (details in Table 2 under Results) was also tested. These included two different *Candida* species, three different isolates of *Bacillus subtilis* and two different isolates each of *Corynebacterium diphtheriae* and *Corynebacterium jeikeium*. Cultures were grown at 37°C in 5% v/v CO_2_ on Lowenstein Jensen (LJ) solid media slants (BD Diagnostics, Sparks, MD) and as described in the ATCC product inserts, except that *Bacillus subtilis* and all *Corynebacterium* species were grown on blood agar plates (Thermo Fisher Scientific, Remel Products, Lenexa, KS). The reference MTBC, two *Bacillus subtilis* (ATCC 23059 and 6633) and the five *Corynebacterium* clinical isolate cultures were grown in the Microbiology Laboratory, University of California San Francisco, San Francisco, CA. Other reference cultures were grown on LJ solid media slants in the ID-FISH laboratory.

**Table 1:** Results of the MN Genus-MTBC and MTBC-MAC FISH assays with reference *Mycobacterium* and *Nocardia* cultures.

Organism	Species	Source	MN Genus	MTBC	MAC
MTBC	*M*. *tuberculosis* H37Rv	ATTC 9360	+	+	-
	*M*. *tuberculosis* RV 37 Ra	ATCC 25177	+	+	-
	*M*. *bovis*	ATCC 19210	+	+	-
	*M*. *bovis—*BCG	ATCC 35734	+	+	-
	*M*. *microti*	ATCC 11152	+	+	-
	*M*. *africanum*	ATCC 25420	+	+	-
MAC	*M*. *avium*	ATCC 25291	+	-	+
	*M*. *avium*	ATCC 35719	+	-	+
	*M*. *avium*	ATCC 19421	+	-	+
	*M*. *avium*	ATCC 700535	+	-	+
	*M*. *avium*	ATCC 25133	+	-	+
	*M*. *avium*	ATCC 15769	+	-	+
	*M*. *avium*	ATCC 35717	+	-	+
	*M*. *avium*	ATCC 19074	+	-	+
	*M*. *avium*	ATCC 29555	+	-	+
	*M*. *avium*	ATCC 35718	+	-	+
	*M*. *avium*	ATCC 49601	+	-	+
	*M*. *intracellulare*	ATCC 35847	+	-	+
	*M*. *intracellulare*	ATCC 13950	+	-	+
	*M*. *scrofulaceum*	ATCC 19981	+	-	+
Other NTM	*M*. *abscessus*	ATCC 19977	+	-	-
	*M*. *bohemicum* BAA	ATCC BAA 2097	+	-	-
	*M*. *branderi*	ATCC 51788	+	-	-
	*M*. *chelonae*	ATCC 35752	+	-	-
	*M*. *conspicuum*	ATCC 700090	+	-	-
	*M*. *fortuitum*	ATCC 6841	+	-	-
	*M*. *fortuitum*	ATCC 13756	+	-	-
	*M*. *gordonae*	ATCC 14470	+	-	-
	*M*. *hiberniae*	ATCC 49874	+	-	-
	*M*. *houstonense*	ATCC 49403	+	-	-
	*M*. *immunogenum*	ATCC 700505	+	-	-
	*M*. *kansasii*	ATCC 12478	+	-	-
	*M*. *lentiflavum*	ATCC 51985	+	-	-
	*M*. *mucogenicum*	ATCC 49650	+	-	-
	*M*. *neworleanense*	ATCC 49404	+	-	-
	*M*. *saskatchewanense*	ATCC BAA544	+	-	-
	*M*. *septicum*	ATCC 700731	+	-	-
	*M*. *shimoidei*	ATCC 49773	+	-	-
	*M*. *simiae*	ATCC 25275	+	-	-
	*M*. *smegmatis*	ATCC 19420	+	-	-
	*M*. *szulgai*	ATCC 35799	+	-	-
	*M*. *triplex*	ATCC 700071	+	-	-
	*M*. *wolinskyi*	ATCC 700010	-	-	-
	*M*. *xenopi*	ATCC 19250	+	-	-
	*Nocardia brasiliensis*	ATCC 19296	+	-	-
Nocardia	*Nocardia nova*	ATCC 33726	+	-	-
	*Nocardia otitidiscaviarum*	ATCC 14629	+	-	-

**Table 2:** Results of the MN Genus-MTBC and MTBC-MAC FISH assays on reference cultures of pathogens not belonging to the *Mycobacterium* or *Nocardia* genera.

Reference Strain	Source	MN Genus	MTBC	MAC
*Bacillus subtilis*	ATCC 23059	**-**	**-**	**-**
*Bacillus subtilis*	ATCC 6633	**-**	**-**	**-**
*Bacillus subtilis*	ATCC 465	-	-	-
*Borrelia burgdorferi*	ATCC 35210	-	-	-
*Burkholderia cepacia*	ATCC 25416	-	-	-
*Candida albicans*	ATCC 18804	-	-	-
*Candida glabrata*	ATCC 2001	-	-	-
*Actinomadura pelleteri*	ATCC 33385	-	-	-
*Arcanobacterium pyogenes*	ATCC 19411	-	-	-
*Corynebacterium amycolatum*	UCSF clinical isolate	-	-	-
*Corynebacterium urealyticum*	UCSF clinical isolate	-	-	-
*Corynebacterium striatum*	UCSF clinical isolate	-	-	-
*Corynebacterium jeikeium*	UCSF clinical isolate	+	-	-
*Corynebacterium diphtheriae*	UCSF clinical isolate	+	-	-
*Corynebacterium diphtheriae*	ATCC 11913	+	-	-
*Corynebacterium jeikeium*	ATCC 43734	+	-	-
*Enterococcus faecalis*	ATCC 19433	-	-	-
*Escherichia coli*	ATCC 25922	-	-	-
*Pseudomonas aeruginosa*	ATCC 27853	-	-	-
*Rhodococcus obuensis*	ATCC 33610	-	-	-
*Staphylococcus aureus*	ATCC 25923	-	-	-
*Staphylococcus haemolyticus*	ATCC 29968	-	-	-
*Staphylococcus lugdunensis*	ATCC 43809	-	-	-
*Streptococcus equi*	ATCC 33398	-	-	-
*Streptococcus salivarius*	ATCC 7073	-	-	-

### Clinical samples

A total of 248 cultures derived from clinical mycobacterial samples were tested. These comprised 174 samples cultured on LJ solid media slants obtained from the Kasturba Medical College, Mangalore, India, the Laboratorio de Investigación en Enfermedades Infecciosas of the Universidad Peruana Cayetano Heredia, Iquitos, Peru and the Microbiology Laboratory at the University of California San Francisco, CA, as well as 74 liquid cultures in Mycobacteria Growth Indicator Tubes (MGIT, BD Diagnostics, Sparks, MD) from the Kasturba Medical College, Mangalore, India. All the samples originated from digested, decontaminated sputum from patients suspected to have tuberculosis. Additionally, phenol-ethanol fixed smears prepared from solid cultures of seven clinical isolates of *Nocardia* identified as *N*. *brasiliensis*, *N*. *farcinica*, *N*. *wallacei*, *N*. *cyriacigeorgica*, *N*. *pseudobrasiliensis*, *N*. *nova* and *N*. *otitidiscaviarum* were obtained from Banner Health, Phoenix, AR.

### Preparation of smears from solid cultures on microscope slides for FISH assays

A loopful of colony was first transferred from the solid medium into distilled water to yield a McFarland standard of 0.5 to 1.0. Several sterile 3 mm glass beads were added to the mixture which was then vortexed for 30 s to facilitate homogenization of the cell suspension. Ten μl of the cell suspension was pipetted onto a pre-cleaned glass microscope slide to make a 15 mm-diameter smear. Smears were then air dried and chemically fixed with either 100% methanol for all samples from Peru and the USA, or a solution of 5% v/v phenol in 70% v/v ethanol in the case of samples from India, for 5 min according to the established procedures in the respective laboratories. This was followed by a rinse with water and air-drying. Fixation in 5% v/v phenol in 70% v/v ethanol renders the fixed smears non-infectious and safe for handling outside a biosafety facility [22].

### Preparation of smears from liquid cultures on microscope slides for FISH assays

Liquid cultures were grown at the Kasturba Medical College, Mangalore, India by inoculating either processed sputum samples or stock cultures into MGIT tubes and placed in the BACTEC ^TM^ MGIT ^TM^ 320 detection system (Becton Dickinson, Franklin Lakes, NJ). Smears for microscopy were prepared usually within a day of a positive signal being detected in the BACTEC ^TM^ MGIT ^TM^ 320 machine. The presence of acid-fast staining bacteria was confirmed prior to preparing smears. The *Mycobacterium* Processing Kit (catalogue number MycoProK04, ID-FISH Technology, Palo Alto, CA) was used to process the liquid cultures according to the manufacturer’s instructions provided in the kit. The kit contained two reagents, a sample processing solution containing a strong reducing agent and MycoSPR containing a chaotropic agent, that help retain optimal specimen morphology in the FISH assay. In the first step, 40 μl of the undiluted culture was added to 40 μl sample processing solution provided in the kit, mixed and incubated for 5–10 min at room temperature, followed by the addition of 10 μl of MycoSPR. Ten μl of the resulting mixture was placed on a pre-cleaned glass microscope slide to generate a 15mm-diameter circular smear. The smears were dried and then fixed with a solution of 5% v/v phenol in 70% v/v ethanol for 5 min at room temperature.

### FISH test kits and reagents

The *Mycobacterium*/*Nocardia* Genus (MN Genus) and MTBC combination FISH test kit (MN Genus-MTBC FISH, catalogue number MycoGTK04) and the MTBC and MAC combination FISH test kit (MTBC-MAC FISH, catalogue number MycoTAcK04) were obtained from ID-FISH Technology Inc., Palo Alto, CA. The two test kits contained the relevant rRNA-specific DNA probes, pretreatment buffer, pretreatment rinse buffer, hybridization solution, wash buffer and mounting medium. The MN Genus-specific probe is complementary to a 16S rRNA sequence that is common to the *Mycobacterium* and *Nocardia* genera. The MTBC and MAC-specific probes hybridize to 23S rRNA sequences that are unique to each of the two species complexes. The tests were performed according to the manufacturer’s instructions provided with the kits.

In the MN Genus-MTBC FISH assay, the MN Genus-specific DNA probe is conjugated with a green fluorescent dye Atto 480 and the MTBC-specific DNA probe is conjugated with an orange fluorescent dye Atto 550. In the MTBC-MAC FISH test, the MTBC-specific DNA probe is conjugated with Atto 480 and the MAC-specific DNA probe is conjugated with Atto 550.

### FISH assay procedure

All FISH assays were performed in the ID-FISH laboratory in Palo Alto, CA, USA. Two slides from each sample were processed with one slide used for the MN Genus-MTBC FISH assay and the other for the MTBC-MAC FISH assay. After the addition of 1 ml of pre-treatment buffer to each fixed smear, the slides were placed in a humid chamber at 37°C for 15 min. The slides were then removed from the incubator, rinsed with pre-treatment rinse buffer and air-dried. After the subsequent addition of 10 μl of appropriate hybridization solution containing the DNA probe mix, the smear was covered with a plastic cover-slip and placed in a 37°C humid chamber for 15 min for hybridization. After hybridization, each smear was washed three times for 2 min each with wash buffer at room temperature and then dried in complete darkness. A drop of mounting medium was then added and the smear was covered with a glass cover-slip. Smears were read at 1000x magnification using a LED light source with custom filter sets for viewing green (excitation 490 nm; emission 529–530nm band pass filter) and orange fluorescence (excitation 530 nm; emission 575 nm long pass filter) obtained from Fraen Corp, Cusago, Italy, fitted to an Olympus BX light microscope. A workflow diagram for performing the FISH assay is provided in S1 Fig. A laboratory microscope with a LED light source and filter attachment (ID-FISH Technology, Palo Alto, CA) is shown in S2 Fig. The patterns of fluorescence expected in the two dual-fluorescence FISH tests with different types of clinically relevant mycobacteria and *Norcardia* are summarized in Table 3.

**Table 3:** Expected Results with *Mycobacterium* and *Norcardia* Cultures in the MN Genus-MTBC and MTBC-MAC FISH Assays.

Cultures	MN Genus—MTBC FISH assay		MTBC—MAC FISH assay	
	MN Genus probe	MTBC probe	MTBC probe	MAC probe
	(green fluorescence)	(orange fluorescence)	(green fluorescence)	(orange fluorescence)
**MTBC**	**+**	**+**	**+**	**-**
**MAC**	**+**	**-**	**-**	**+**
**Non-MAC NTM**	**+**	**-**	**-**	**-**
**Mix of MTBC and MAC**	**+**	**+**	+ (reaction only with MTBC cells)	+ (reaction only with MAC cells)
***Nocardia***	**+**	**-**	**-**	**-**

### Sequencing of 23S rDNA from clinical mycobacterial isolates

A sample was taken from a smear made from each of the 248 mycobacterial cultures and processed to obtain purified DNA using the Generation Capture Column kit (Qiagen, Valencia, CA). The relevant DNA region was then amplified by PCR using the following forward and reverse primers: Myco-F, 5’-AGAATGAGCCTGCGAGTCAG-3’ and Myco-R, 5’-ACARCTCATCCCCTCAGTCT-3’. The approximately 200bp PCR products were subsequently sequenced using the same Myco-F and Myco-R primers. The resulting sequences were compared by BLAST with published *Mycobacterium* 23S rDNA sequences to identify the mycobacterial species.

### Determination of the limit of detection (LOD) in the FISH assays

With an approximate starting concentration of 10^6^ to 10^7^ cells per ml from MGIT cultures, serial ten-fold dilutions in MGIT broth up to a dilution of 10^−7^ were prepared from three different replicate *M*. *tuberculosis* (ATCC 25177) and three different replicate *M*. *avium* (ATCC 25291) cultures. At each dilution, 10 μl samples were plated on Middlebrook 7H10 agar plates and incubated at 37°C, 5% v/v CO_2_ for up to four weeks. Colonies were counted after sufficient growth was obtained to determine the number of colony forming units (cfu) at each dilution. Three replicate smears prepared in parallel from each of the same dilutions were tested in both MN Genus-MTBC FISH and MTBC-MAC FISH assays. Three hundred fields were examined at 1000x magnification before recording a negative reaction for a smear in the limit of detection (LOD) determination. The LOD was defined as the lowest concentration of bacteria at which the three replicate smears for each original culture tested gave a positive result with relevant probes in the two FISH assays. The final LOD was calculated as the mean of the three LODs derived for the three replicate starting cultures for each species.

### Data analysis

The diagnostic sensitivity, specificity, positive predictive value and negative predictive value and their 95% confidence intervals were calculated using an online statistical calculator [23] for each probe in the two FISH assays. The accuracy, defined as the proportion of test samples that were correctly identified, was also determined for each probe.

## Results

### Results of the two FISH assays on cultures of clinically relevant reference pathogens

**Fig 1:**
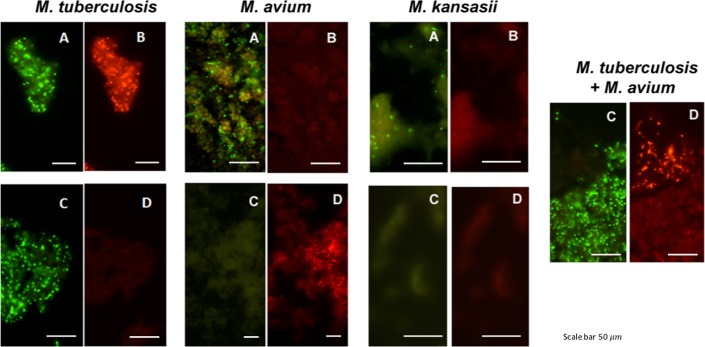
Photographs showing the dual color fluorescence reactivity of individual cultures of *Mycobacterium tuberculosis*, *Mycobacterium avium* and *Mycobacterium kansasii* in the MN Genus-MTBC FISH and MTBC-MAC FISH assays and of a mixed culture of *Mycobacterium tuberculosis* and *Mycobacterium avium* in the MTBC-MAC FISH assay. A, B–Dual fluorescence reactivity in the same microscopic field with the MN Genus- and MTBC-specific probes respectively in the MN Genus-MTBC FISH assay; C, D—Dual fluorescence reactivity with the MTBC- and MAC-specific probes respectively in the MTBC-MAC FISH assay. Mycobacteria used in the FISH tests were: LJ culture of *M*. *tuberculosis* (ATCC 25177); LJ culture of *M*. *avium* (ATCC 25291); MGIT culture of *M*. *kansasii* (ATCC 12478); and an artificially mixed LJ culture of *M*. *tuberculosis* (ATCC 25177) and *M*. *avium* (ATCC 25291). Photographs were taken at x1000 magnification. Scale bars shown in the photographs represent approximately 50 *μm*.

The dual color fluorescence observed in the two FISH assays with individual culture smears of reference strains of *M*. *tuberculosis*, *M*. *avium* and *M*. *kansasii*, and with the MTBC-MAC FISH test on a smear from an artificially mixed culture of reference strains of *M*. *tuberculosis* and *M*. *avium* are shown in Fig 1. The dual color fluorescence seen in each case is consistent with the expected outcome shown in Table 3.

Table 1 shows that all 44 reference *Mycobacterium* species cultures, except for *M*. *wolinskyi*, tested positively with the MN Genus-specific probe and only the six *M*. *tuberculosis* complex cultures reacted positively with the MTBC- specific probe in the MN Genus—MTBC FISH assay. Of the same 44 *Mycobacterium* species cultures tested in the MTBC-MAC FISH assay, only the six *M*. *tuberculosis* complex cultures reacted positively with the MTBC-specific probe and only the 14 *M*. *avium* complex cultures reacted positively with the MAC–specific probe (Table 1). All three *Nocardia* reference cultures tested reacted positively with the MN Genus probe but not with the MTBC- and MAC-specific probes in the MN Genus-MTBC and the MTBC-MAC assays respectively (Table 1).

Of the 25 other clinically relevant reference pathogen cultures tested that did not belong to either the genus *Mycobacterium* or the genus *Nocardia*, 21 did not react with the MN Genus-specific probe (Table 2). Two different isolates each of *Corynebacterium diphtheriae* and *Corynebacterium jeikeium* however reacted positively with the MN Genus-specific probe while three other corynebacterial species, *viz*. *Corynebacterium amycolatum*, *Corynebacterium striatum* and *Corynebacterium urealyticum* did not react with the same probe (Table 2). All 25 of the non-*Mycobacterium*/non-*Nocardia* species tested were negative with the MTBC- and MAC- specific probes in the two FISH assays (Table 2).

S2 Table shows details of the estimates of the sensitivity, specificity, positive and negative predictive values, and accuracy for the MN Genus-, MTBC- and MAC-specific probes in the FISH assays with the 72 reference cultures listed in Tables 1 and 2. The results show that the MTBC and MAC probes are estimated to be 100% accurate. The MN Genus probe is estimated to be 93.1% accurate.

### Limits of detection (LOD) in the FISH assays

The LOD for MTB was determined to be 5.1x10^4^ cfu per ml and for *M*. *avium* 1.5x10^4^ cfu per ml in liquid cultures with the respective MTBC- and MAC-specific probes in both the MN Genus-MTBC and MTBC-MAC FISH assays. The LODs with the MN Genus-specific probe in the MN Genus-MTBC FISH assay were also 5.1x10^4^ cfu per ml for MTB and 1.5x10^4^ cfu per ml for *M*. *avium*.

### Application of the FISH assays to clinical mycobacterial isolates from India, Peru and the USA and *Nocardia* isolates from the USA

A total of 248 mycobacterial cultures from LJ slants (174 cultures) and MGITs (74 cultures) were tested in both the MN Genus-MTBC FISH and MTBC-MAC FISH assays. In addition, all cultures were subjected to PCR amplification and sequencing of 23S rDNA. Of the 248 cultures tested in the two FISH assays, 247, 158 and 50 were detected by the MN Genus-, MTBC- and MAC-specific probes respectively. PCR amplification was successful with 243 of the 248 cultures tested (Table 4). In the 243 samples where sequencing was possible, deletions or insertions were not observed in the 23S rDNA regions targeted by the probes used in the FISH assays. Four LJ cultures from Peru and one MGIT culture from India, did not yield PCR-amplified products, and therefore it was not possible to obtain 23S rDNA sequence for definitively identifying these isolates. (Table 4). Four of the PCR-negative samples (three LJ cultures from Peru and one MGIT culture from India) were positive by the MN Genus FISH assay and one (the MGIT culture from India) was also positive for MTBC by FISH. One LJ culture from Peru that did not produce a PCR product proved to be negative in both FISH assays.

**Table 4:** Results of the DNA sequence-based characterization and the MN Genus-MTBC and MTBC-MAC FISH assays on clinical mycobacterial cultures from India, Peru and the USA.

Origin (culture medium)	Total number of samples	Number negative by both FISH and PCR	Number identified as Mycobacteria		Number identified as MTBC		Number identified as MAC		Other non-MAC NTM identified
			MN Genus probe	Both MN Genus probe and DNA sequencing	MTBC probe	Both MTBC probe and DNA sequencing	MAC probe	Both MAC probe and DNA sequencing	Both MN Genus probe and DNA sequencing
India (LJ)	54	0	54	54	42	42	0	0	12 (9 *M*. *abscessus*, 3 *M*. *marinum*)
Peru (LJ)	70	1	69	66	65	65	0	0	1 (*M*. *immunogenum*)
USA (LJ)	50	0	50	50	0	0	47	47	3 *(M*. *immunogenum*)
India (MGIT)	74	0	74	73	51	50	3	3	20 (1 *M*. *smegmatis*, 1 *M*. *marinum*, 1 *M*. *chelonae*, 17 *M*. *abscessus*)
Total	248	1	247	243	158	157	50	50	36

All 157 mycobacterial clinical isolates categorized as MTBC by DNA sequencing were detected with the MTBC-specific probe in both the MN Genus-MTBC FISH and MTBC-MAC FISH assays. Of the remaining 86 mycobacterial clinical isolates characterized by DNA sequencing, 50 were from UCSF, USA where they had been identified as MAC using the commercially available AccuProbe test for MAC (Hologic, Marlborough, MA). However, only 47 of the 50 were confirmed as belonging to MAC by 23S rDNA sequencing in the present study. All these 47 specimens tested positive for MAC in the MTBC-MAC FISH assay. The remaining 3 were negative in the MTBC-MAC FISH assay and were confirmed to be *M*. *immunogenum* by DNA sequencing (Table 4). In total 36 mycobacterial isolates that were negative for MAC and MTBC and positive for MN Genus by FISH, were also identified from their 23S rRNA sequences as belonging to non-MAC NTM. Twenty six of these were identified as *M*. *abscessus*, one as *M*. *chelonae*, four as *M*. *immunogenum*, four as *M*. *marinum*, and one as *M*. *smegmatis* (Table 4). The proportion of such non-MAC NTM was significantly higher in the Indian than Peruvian isolates (Fisher’s exact two-tailed test, P<0.0001).

All seven clinical specimens of *Nocardia* species (*N*. *brasiliensis*, *N*. *farcinica*, *N*. *wallacei*, *N*. *cyriacigeorgica*, *N*. *pseudobrasiliensis*, *N*. *nova and N*. *otitidiscaviarum*) were positive with the MN Genus-specific probe and negative with the MTBC- and MAC-specific probes in the FISH assays, consistent with the expectation summarised in Table 3.

The sensitivity, specificity, positive and negative predictive values, and accuracy for the MN Genus-, MTBC- and MAC-specific probes in both FISH assays were estimated for the 243 *Mycobacterium* clinical isolate cultures that could be identified by DNA sequencing (Table 5). The MN Genus-specific, MTBC-specific and MAC-specific probes were estimated to be 100% sensitive and accurate in detecting *Mycobacterium* genus, MTBC and MAC respectively in the two FISH assays. The specificity of the MN Genus-specific probe for the clinical isolate cultures could not be determined because there were no expected negatives, i.e. non-*Mycobacterium*, non-*Nocardia* samples, among the clinical isolate cultures that were characterized by DNA sequencing. However the specificities of the MTBC- and MAC-specific probes were estimated to be 100%. Details of the calculations are shown in S3 Table.

**Table 5:** Estimated sensitivity, specificity, and positive and negative predictive values of the FISH probes on 243 clinical mycobacterial cultures that could be identified by DNA sequencing.

FISH Probe	Sensitivity (95% CI)	Specificity (95% CI)	PPV (95% CI)	NPV (95% CI)	Accuracy
MN Genus	100% (98.1–100)	N/A	100% (98.1–100)	N/A	100.00%
MTBC	100% (97.0–100)	100% (95.1–100)	100% (97.0–100)	100% (95.1–100)	100.00%
MAC	100% (91.1–100)	100% (97.7–100)	100% (91.1–100)	100% (97.7–100)	100.00%

CI—confidence interval; N/A- not applicable as this could not be determined because there were no true negatives; NPV–negative predictive value; PPV- positive predictive value

## Discussion

The findings suggest that a combination of the MN Genus-MTBC and MTBC-MAC FISH assays can be effective diagnostic tools for detecting *Mycobacteria* from solid and liquid cultures and for their identification as MTBC, MAC or NTM other than MAC. The MN Genus-MTBC and MTBC-MAC FISH assays are best performed sequentially in that order on cultures so that non-MTBC mycobacteria identified in the MN Genus-MTBC assay can subsequently be classified as MAC or other NTM by the MTBC-MAC assay. The MTBC-MAC FISH assay described here is the first molecular test that can detect and differentiate the MTBC and MAC in a single smear. Our findings further suggest that the FISH assays are useful for simultaneously detecting mixed infections of MTBC and MAC. The two FISH assays have a LOD of at least 5.1x10^4^ cfu of bacilli per ml which can help minimize delays in diagnosis by being applicable relatively early after initiation of cultures. The two assays are also rapid, with results becoming available in less than two hours. The likely universal applicability of the MN Genus-MTBC and MTBC-MAC FISH assays is demonstrated by the results with clinical samples from three geographically disperse countries *viz*. India, Peru and the USA.

Only one species of the genus *Mycobacterium* tested, *viz*. *M*. *wolinskyi*, which is a rare clinical isolate and a rapidly growing member of the *M*. *smegmatis* group [24], was missed in the MN Genus FISH assay. Analysis of published sequence suggests that the 16S rDNA target sequence in *M*. *wolinskyi* is different enough to not hybridize to the MN Genus-specific probe. *Nocardia* species are detected only with the MN Genus-specific probe in the MN Genus-MTBC FISH assay, and are not distinguished from non-MAC NTM with the two FISH assays (Table 3). *Nocardia* and non-MAC NTM can however be readily differentiated by colony characteristics, differential acid-fast staining and biochemical methods. Two species of the genus *Corynebacterium viz*. *C*. *diphtheriae* and *C*. *jeikeium* but not three others *viz*. *C*. *amycolatum*, *C*. *striatum* and *C*. *urealyticum* also reacted with the MN Genus probe. *Corynebacterium* is a genus related to *Nocardia* and *Mycobacterium* with the three genera being classified within the order *Actinomycetales*. Analysis of the 16S rDNA sequence that is the target of the MN Genus-specific probe in *C*. *diphtheriae* and *C*. *jeikeium* showed that the sequence differences present would not be sufficient to prevent cross-hybridization of the MN Genus probe. The corresponding 16S rDNA sequences in *C*. *amycolatum*, *C*. *striatum* and *C*. *urealyticum* were not available but it may be hypothesized that these are divergent enough to preclude hybridization of the MN Genus probe. However the cross-reaction with the MN Genus probe implies that some *Corynebacterium* species, like *Norcardia* species, have to be differentiated from non-MAC NTM by other means e.g. clinical presentation, differential acid-fast staining, biochemical methods and culture characteristics.

NTM, particularly MAC, are an important cause of pulmonary disease, especially among HIV-positive populations [3, 4]. The two FISH assays are a useful diagnostic tool in such settings. The higher proportion of non-MAC NTM observed in the clinical mycobacterial samples from India compared to Peru is unexpected and the cause is under further investigation. It is possible that the difference may be due to a higher proportion of the Indian samples originating from immunocompromised patients who are susceptible to infection with non-MAC NTM prevalent in their particular environment.

The technical advantages of using a LED unit with appropriate filters attached to a regular light microscope (S2 Fig) for the FISH assays are that the LED attachment is easy to install, needs little maintenance, able to operate on a rechargeable battery unit for use in remote laboratories, and has a longer lifetime, no decay curve, and is less of a health hazard than a mercury lamp fluorescence microscope.

The FISH assays reported here therefore offer many advantages over other common assays used for characterizing clinical mycobacterial cultures (a summary comparison is presented in S1 Table). Also it may be possible to develop analogous FISH assays for identifying NTM other than MAC at the species level for additional diagnostic purposes. Ongoing studies are investigating the possible application of the two FISH assays described here to sputum smear and tissue samples for detecting MTBC, MAC and other NTM/*Nocardia* in tuberculosis-endemic countries. The WHO recommends that diagnostic testing for tuberculosis should be based on country epidemiology, existing laboratory networks and available resources. In this context improved diagnostic tests suitable for peripheral (community) and intermediate (district and sub-district) level laboratories will help reduce delays in diagnosing and initiating treatment of patients in many tuberculosis-endemic countries. We suggest in conclusion that the FISH assays described here meet this important diagnostic need.

### Ethics

The study was reviewed and approved by the Institutional Review Boards of the Kasturba Medical College, Mangalore, India; Johns Hopkins Bloomberg School of Public Health, Bethesda, MA; Universidad Peruana Cayetano Heredia, and Asociación Benéfica PRISMA, both in Lima, Peru; and the Directorate of Health, Iquitos, Peru. Approval was granted to use archived de-identified clinical samples that would otherwise be discarded. Patient consent was not required, since cultures used in the study were only derived from left-over de-identified patient sputum samples submitted for routine testing, that would otherwise have been discarded.

## Supporting information

S1 Fig Workflow Chart for the FISH Assays. (PDF)

S2 Fig Microscope with LED and Filter Attachment. (PDF)

S1 Table Comparison of the FISH Assays with Three Common Molecular Tests for Identifying Cultured Mycobacteria. (PDF)

S2 Table Estimates of Sensitivity, Specificity, Predictive Values, and Accuracy of the FISH Probes with Reference Pathogen Cultures. (PDF)

S3 Table Estimates of Sensitivity, Specificity, Predictive Values, and Accuracy of the FISH Probes with Clinical Mycobacterial Cultures. (PDF)
