# Supplementary figures and images for: Dual color fluorescence in situ hybridization (FISH) assays for detecting Mycobacterium tuberculosis and Mycobacterium avium complexes and related pathogens in cultures
Source: PLoS One. 2017 Apr 11;12(4):e0174989. doi: 10.1371/journal.pone.0174989 (PMC5388335; doi:10.1371/journal.pone.0174989)

### **S1Fig. Workflow Chart for the FISH Assays**

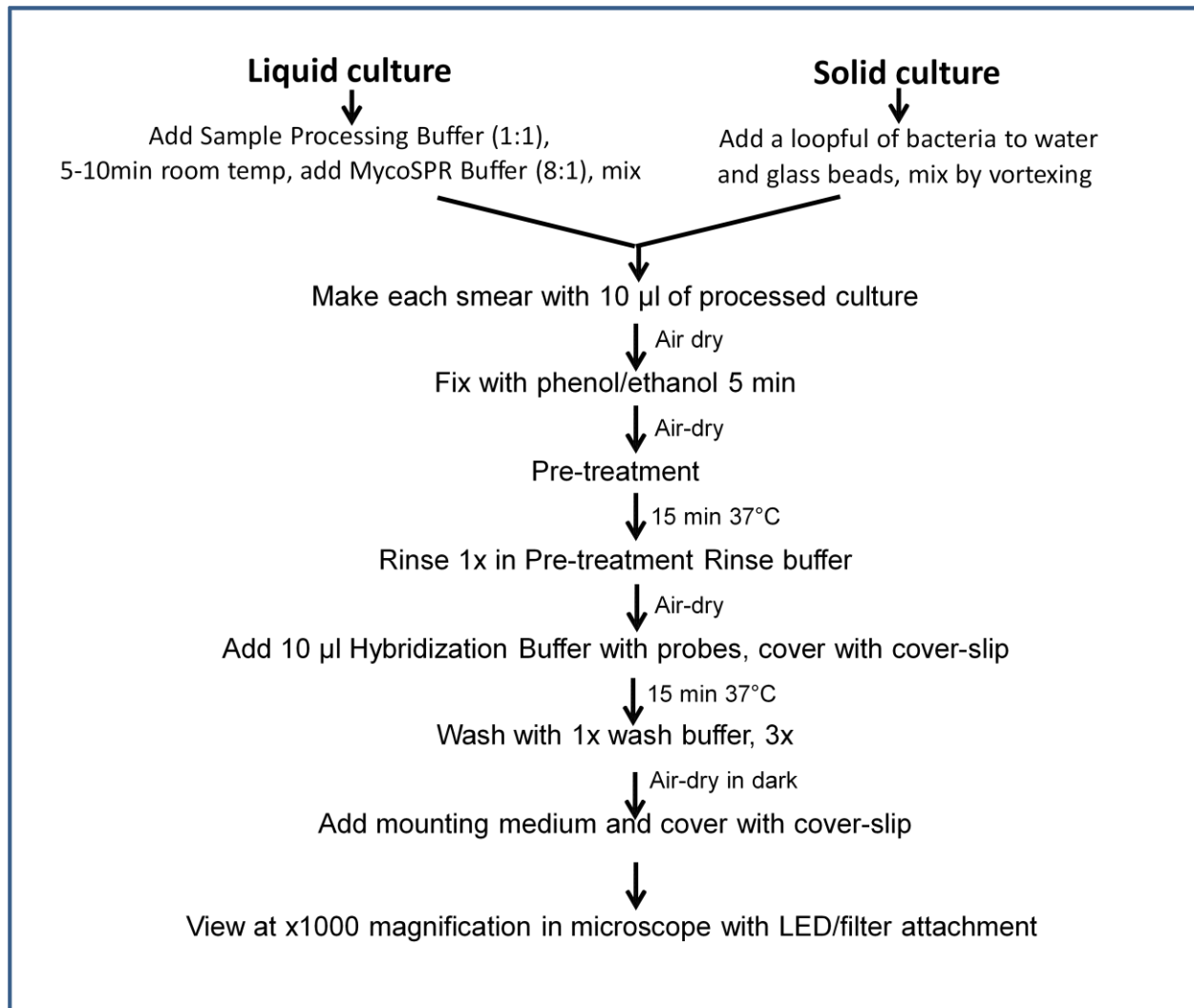

Supplement: S1 Fig — (PDF) [file pone.0174989.s001.pdf]

**S2 Fig. Microscope with LED and Filter Attachment**

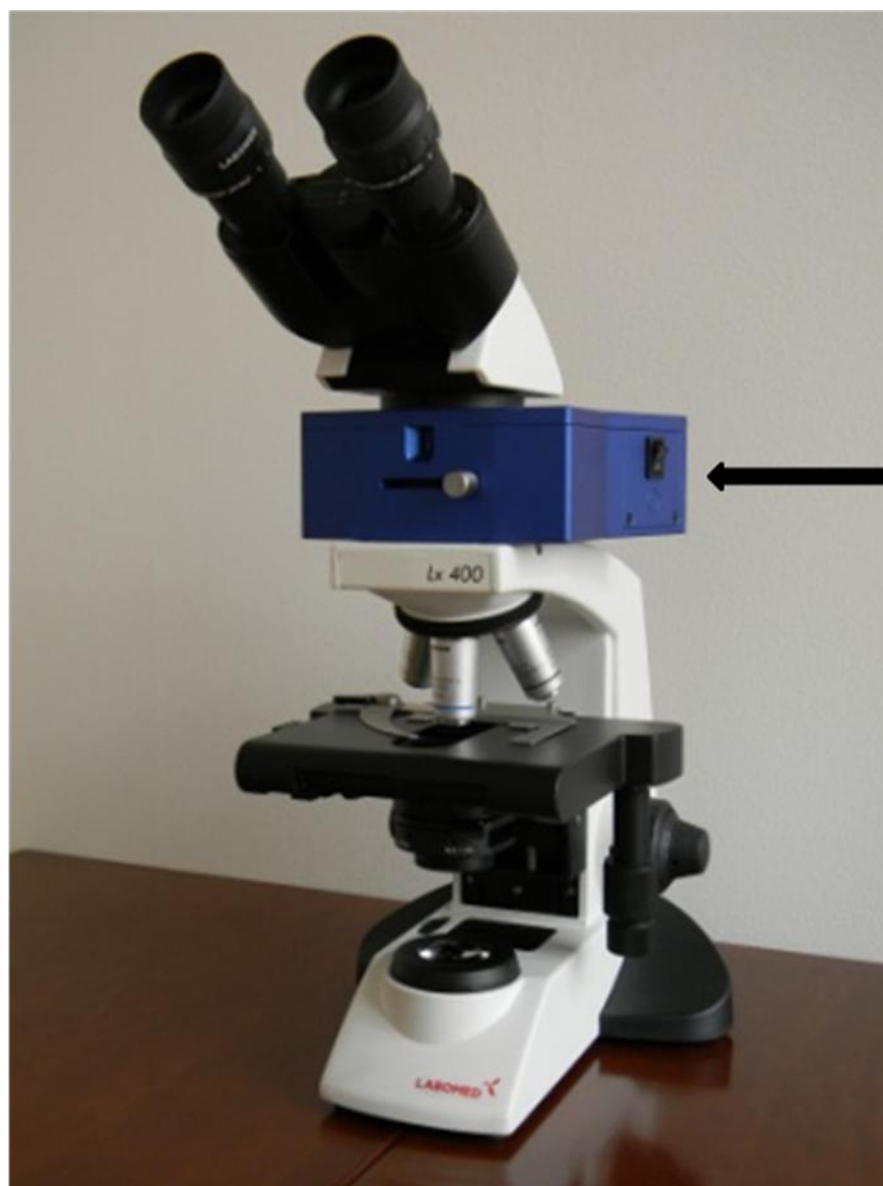

LED and Filter  
Attachment

Supplement: S2 Fig — (PDF) [file pone.0174989.s002.pdf]
