# Supplementary material for: Dual color fluorescence in situ hybridization (FISH) assays for detecting Mycobacterium tuberculosis and Mycobacterium avium complexes and related pathogens in cultures
Source: PLoS One. 2017 Apr 11;12(4):e0174989. doi: 10.1371/journal.pone.0174989 (PMC5388335; doi:10.1371/journal.pone.0174989)
Supplement: S1 Table — (PDF) [file pone.0174989.s003.pdf]

S1 Table. Comparison of the FISH Assays with Three Common Molecular Tests for Identifying Cultured Mycobacteria

Test (Supplier)	Detection Principle	Expected Output	Performance Characteristics
AccuProbe (Hologic, Marlborough, MA)	1. Chemiluminescent probes hybridizing to rRNA and then detection of bound luminescence in a luminometer.<br>2. Applied to solid and liquid cultures.<br>3. Acid-fast stain positive cultures are first tested with an Accu Probe MTBC-specific assay. MTBC –ve cultures are subsequently tested with AccuProbes specific for *M. avium*, and *M. avium*, *M. intracellulare*, *M. gordonae* and *M. kansasii* complexes.	Identification of the major mycobacterial species complexes.	1. Results in a few hours.<br>2. Starting McFarland standard of ≥1, *i.e.* ≈≥$3x10^8$ cfu/ml is recommended by manufacturer for liquid cultures.<br>3. Sensitivity reported to approach 100%. Issues reported with specificity of MTBC [1, 2] and MAC tests [3, our data in manuscript].<br>4. Refrigerated storage needed for probes.<br>5. Relatively expensive.<br>6. Commonly used in the USA.
INNO-LiPA® Mycobacteria test (Fujirebio, Gent, Belgium)	1. PCR amplification of the 16S - 23S rDNA spacer sequence and detection of binding of the amplified, biotinylated DNA to a linear array of probes in a single test strip. Labelled bands on strip as readout.<br>2. Applicable to solid and liquid cultures.<br>3. Test done on acid-fast stain positive cultures.	Specific identification of *Mycobacterium* genus, MTBC and MAC and several other mycobacterial species. Mixed populations identifiable.	1. Relatively labor intensive and time consuming.<br>2. Good LOD as test is based on PCR amplification.<br>3. Sensitivity of 98.8% and specificity of 100% reported for *Mycobacterium* genus detection.<br>4. Refrigeration of reagents required.<br>5. Relatively expensive.<br>6. Used outside the USA to detect and differentiate MTBC and MAC.
BD MGIT™ TBc identification test (	1. Immunochromatographic	Specific identification of MTBC.	1. Rapid ≈ 15 min.

Becton Dickinson Diagnostics, Sparks, MA)	detection of the MPB64 (MPT64) protein produced by MTBC in liquid cultures. Labelled band on strip as readout.<br>2. Applied to liquid cultures.<br>3. Test done on acid-fast stain positive cultures.		2. LOD reported as $5x10^5$ cfu per ml [4].<br>3. Issues with specificity due to cross reaction with NTM [5, 6, 7] and sensitivity due to low or null transcription of *mpb64* gene [5, 8, 9].<br>4. Refrigeration of reagents required.<br>5. Relatively inexpensive.<br>6. Easy to perform, and suitable for resource-limited countries.
MN Genus – MTBC and MTBC-MAC FISH assays (IDFISH Technology Inc., Palo Alto, CA.	1. Hybridization of DNA probes labelled with fluorophores to specific rRNA target sequences in *Mycobacterium/Nocardia* genus, MTBC and MAC. Use of light microscope with an LED/filter attachment as alternative to fluorescence microscope feasible.<br>2. Applicable to liquid and solid cultures.<br>3. Test done on acid-fast stain positive cultures.	Specific identification of *Mycobacterium/Nocardia* genus, MTBC and MAC. Mixed populations of MTBC and NTM identifiable. Non-MAC NTM and *Nocardia* suggested by reaction with the MN Genus probe and not with the MTBC and MAC probes. Non-MAC NTM and *Nocardia* need distinguishing by culture characteristics, differential acid-fast staining and other methods.	1. Results in <2h.<br>2. LOD $\leq 5.1x\ 10^4$ cfu/ml.<br>3. Accuracy of 100% with clinical mycobacterial isolates.<br>4. Assay kits stable at up to 30^0C for several months.<br>5. Relatively inexpensive.<br>6. Easy to perform and suitable for resource-limited countries.

References Cited in Supplementary S1Table

1. Ford EG, Snead SJ, Todd J, Warren NG. Strains of Mycobacterium terrae complex which react with DNA probes for M. tuberculosis complex. J Clin Microbiol. 1993; 31:2805–2806.
2. Butler WR, O'Connor SP, Yakrus MA, Gross WM. Cross-reactivity of genetic probe for detection of Mycobacterium tuberculosis with newly described species Mycobacterium celatum. J Clin Microbiol. 1994; 32:536–538.

3. Cloud JL, Carroll KC, Cohen S, Anderson CM, Woods GL. Interpretive criteria for use of AccuProbe for identification of Mycobacterium avium complex directly from 7H9 broth cultures. J Clin Microbiol. 2005; 43:3474-3478.
4. Yu MC, Chen HY, Wu MH, Huang WL, Kuo YM, Yu FY et al. Evaluation of the rapid MGIT TBc identification test for culture confirmation of Mycobacterium tuberculosis complex strain detection. J Clin Microbiol. 2011; 49: 802-807.
5. Brent AJ, Mugo D, Musyimi R, Mutiso A, Morpeth S, Levin M et al. Performance of the MGIT TBc identification test and metaanalysis of MPT64 assays for identification of the Mycobacterium tuberculosis complex in liquid culture. J Clin Microbiol. 2011; 49:4343-4346. doi: 10.1128/JCM.05995-11.
6. Hasegawa N, Miura T, Ishii K, Yamaguchi K, Lindner TH, Merritt S et al. New simple and rapid test for culture confirmation of Mycobacterium tuberculosis complex: a multicenter study. J Clin Microbiol. 2002; 40: 908–912.
7. Martin A, Bombeeck D, Fissette K, de Rijk P, Hernández-Neuta I, Del Portillo P et al. Evaluation of the BD MGIT TBc Identification Test (TBc ID), a rapid chromatographic immunoassay for the detection of Mycobacterium tuberculosis complex from liquid culture. J Microbiol Methods. 2011; 84:255-7. doi: 10.1016/j.mimet.2010.12.003..
8. Hirano K, Aono A,Takahashi M, Abe C. Mutations including IS6110 insertion in the gene encoding the MPB64 protein of Capilia TB negative Mycobacterium tuberculosis isolates. J Clin Microbiol. 2004; 42:390– 392.
9. Muyoyeta, M. Evaluation of the Capilia TB assay for culture confirmation of Mycobacterium tuberculosis infections in Zambia and South Africa. J Clin Microbiol. 2010; 48:3773–3775.
