# Supplementary material for: Dual color fluorescence in situ hybridization (FISH) assays for detecting Mycobacterium tuberculosis and Mycobacterium avium complexes and related pathogens in cultures
Source: PLoS One. 2017 Apr 11;12(4):e0174989. doi: 10.1371/journal.pone.0174989 (PMC5388335; doi:10.1371/journal.pone.0174989)
Supplement: S2 Table — (PDF) [file pone.0174989.s004.pdf]

S2 Table. Estimates of Sensitivity, Specificity, Predictive Values, and Accuracy of the FISH Assays with Reference Pathogen Cultures

MN Genus Probe						
					(%)	95% Confidence Interval
		Expected		Sensitivity	97.9	87.3-99.9%
		(+)	(-)	Specificity	84.0	63.1-94.7
FISH	(+)	46	4	PPV	92.0	79.9-97.4
	(-)	1	21	NPV	95.5	75.1-99.8

Accuracy 93.1%

MTBC Probe						
					(%)	95% Confidence Interval
		Expected		Sensitivity	100.0	51.7-100%
		(+)	(-)	Specificity	100.0	93.1-100
FISH	(+)	6	0	PPV	100.0	51.7-100
	(-)	0	66	NPV	100.0	93.1-100

Accuracy 100%

MAC Probe						
					(%)	95% Confidence Interval
		Expected		Sensitivity	100.0	73.2 - 100%
		(+)	(-)	Specificity	100.0	92.3 - 100
FISH	(+)	14	0	PPV	100.0	73.2 - 100
	(-)	0	58	NPV	100.0	92.3 - 100

Accuracy 100%

PPV – Positive predictive value; NPV – Negative predictive value.
