# Supplementary material for: Dual color fluorescence in situ hybridization (FISH) assays for detecting Mycobacterium tuberculosis and Mycobacterium avium complexes and related pathogens in cultures
Source: PLoS One. 2017 Apr 11;12(4):e0174989. doi: 10.1371/journal.pone.0174989 (PMC5388335; doi:10.1371/journal.pone.0174989)
Supplement: S3 Table — (PDF) [file pone.0174989.s005.pdf]

S3 Table. Estimates of Sensitivity, Specificity, Predictive Values, and Accuracy of the FISH Assays with Clinical Mycobacterial Cultures

MN Genus Probe						
					(%)	95% Confidence Interval
		Expected		Sensitivity	100.0	98.1 - 100%
		(+)	(-)	Specificity	N/A	N/A
FISH	(+)	243	0	PPV	100.0	98.1 - 100
	(-)	0	0	NPV	N/A	N/A

Accuracy 100%

MTBC Probe						
					(%)	95% Confidence Interval
		Expected		Sensitivity	100.0	97.0 - 100%
		(+)	(-)	Specificity	100.0	94.7 - 100
FISH	(+)	157	0	PPV	100.0	97.0 - 100
	(-)	0	86	NPV	100.0	94.7 - 100

Accuracy 100%

MAC Probe						
					(%)	95% Confidence Interval
		Expected		Sensitivity	100.0	91.1 - 100
		(+)	(-)	Specificity	100.0	97.6 - 100
FISH	(+)	50	0	PPV	100.0	91.1 - 100
	(-)	0	193	NPV	100.0	97.6 - 100

Accuracy 100%

Specificity and NPV for the MN Genus probe could not be determined as there were no true negatives

PPV = Positive predictive value

NPV= Negative predictive value

N/A = Not applicable
